# Supplementary material for: Celiac Disease and the Gluten Free Diet during the COVID-19 Pandemic: Experiences of Children and Parents
Source: Medicina (Kaunas). 2023 Feb 21;59(3):425. doi: 10.3390/medicina59030425 (PMC10059717; doi:10.3390/medicina59030425)
Supplement: Supplementary file 1 [file medicina-59-00425-s001.zip › Questionnare celiac disease and Covid-19 lockdown; children younger than 12 years S1.pdf]

medicina

MDPI

S1 Questionnaire

Celiac disease and the COVID-19 pandemic

Children younger than 12 years

Content

Complaints 2

Complaints family members 3

COVID-19 measures 4

The gluten free diet 5

You and the gluten free diet 5

The gluten free diet during the COVID-19 pandemic 6

Nutrition before implementation of COVID-19 measures 8

Nutrition during COVID-19 pandemic 10

Length and weight 13

Complaints

This questionnaire was designed to gain insight into whether your child has had complaints since the start of the covid-19 outbreak that could be associated with an infection with an infection with the covid-10 virus or that could be consistent with celiac disease or following the gluten-free diet. For this we would like to know what complaints your child has developed since the start of the carnival holiday (22-02-2020).

NOTE: These answers are only used for research and not do diagnose disease. Do you or a family member have complaints and are you worried? Then contact your own family doctor.

1. Has your child developed new complaints in the period since the start of the carnival holiday (22-02-2020) or have the following complaints become more severe? Select the complaints that have developed or that have become worse than normal.
   - ☐ Common cold
   - ☐ Coughing
   - ☐ Stuffy feeling
   - ☐ Shortness of breath or wheezing
   - ☐ Sore throat
   - ☐ Fever (body temperature >38.0°C)
   - ☐ Sneezing
   - ☐ Coughing up mucus
   - ☐ Fatigue
   - ☐ Muscle soreness
   - ☐ Chest pain
   - ☐ Diarrhea
   - ☐ Stomach ache
   - ☐ Nausea/ vomiting
   - ☐ Eye infection
   - ☐ Lost sense of smell
   - ☐ Headache
   - ☐ Constipation
   - ☐ Bloated stomach/ flatulence
   - ☐ Weight loss
   - ☐ Lower energy level
   - ☐ Red itchy rash
   - ☐ Sores in mouth
2. Have you contacted your general practitioner or pediatrician regarding complaints that your child experienced that resemble a corona infection.
   a. ☐ Yes
   b. ☐ No
3. Has your child been tested to determine whether he / she was infected by the corona virus? (By test we mean a sample of saliva, a throat swab or CT scan)
   a. ☐ Yes
   b. ☐ No

If yes, which test was performed?……………………………………………………………………………………………

4. What was the result of this test?
   a. ☐ Positive (corona infection established)
   b. ☐ Negative

5. Was your child admitted to a hospital for a possible infection with the corona virus?
   a. ☐ Yes
   b. ☐ No

If yes, how many days has he/she been admitted?

………………………………………………………………………………

If yes, how did the admission go?

……
……
……
……
……
………………………………………………………………………………

Complaints family members

We also want to map whether anyone in the family has been ill since the start of the Carnival holiday (22-02-2020). This concerns all people who live in the household. Are parents no longer together and does your child live in both families? Then fill in the complaints below for all family members from both households.

6. Has a member of the family or someone who lives in your household(s) developed **new complaints** or have the following complaints become **more severe** since the start of the Carnival holiday (22-02-2020)?
   a. ☐ Yes
   b. ☐ No

   If no, you may continue to question 7.

Witch family members have developed new complaints or have experienced more severe complaints from the complaints listed below?

   a. ☐ Mother
   b. ☐ Father
   c. ☐ Brother/sister (cross out if not applicable) age: ………………………..…
   d. ☐ Brother/sister (cross out if not applicable) age: ……………………….
   e. ☐ Other family member namely: ….…………..…., age: ………………

For each family member, tick which complaints have developed new or have become worse than normal:

	Mother	Father	Brother/sister	Brother/sister	Other family member
Common cold	☐	☐	☐	☐	☐
Coughing	☐	☐	☐	☐	☐
Stuffy feeling	☐	☐	☐	☐	☐
Shortness of breath or wheezing	☐	☐	☐	☐	☐
Sore throat	☐	☐	☐	☐	☐
Fever	☐	☐	☐	☐	☐
Sneezing	☐	☐	☐	☐	☐
Coughing up mucus	☐	☐	☐	☐	☐
Fatigue	☐	☐	☐	☐	☐
Muscle soreness	☐	☐	☐	☐	☐
Chest pain	☐	☐	☐	☐	☐
Diarrhea	☐	☐	☐	☐	☐
Stomach ache	☐	☐	☐	☐	☐
Nausea/ vomiting	☐	☐	☐	☐	☐
Eye infection	☐	☐	☐	☐	☐
Lost sense of smell	☐	☐	☐	☐	☐
Headache	☐	☐	☐	☐	☐

7. Has a family member been tested to determine whether an infection with the corona virus was present? (By test we mean a sample of saliva, a throat swab or CT scan)
   a. ☐ Yes
   b. ☐ No

If yes, which test? …….

If yes, how many family members have been tested?……………………………………………………………..

8. What was the result of this test?
   a. ☐ Positive (corona infection established)
   b. ☐ Negative

If several family members have been tested with different results, please explain here.

………
………
………
……………………..

9. Was a family member admitted to hospital for a possible infection with the corona virus?
   a. ☐ Yes
   b. ☐ No

   If yes, how many days has he/she been admitted?

………………………………………………………………………
If yes, how did the admission go?
……
……
……
………………………………………………..

COVID-19 measures

Since March 23th, strict measures have been applied to contain the spread of the corona virus. The following questions are about your family's compliance with these measures. The data from this research is confidential and will not be distributed further. We therefore ask you to answer the questions as honestly as possible. Depending on the age, some measures do not apply to your child.

10. To what extent is your family complying with the following measures (circle if applicable)

Wash your hands regularly with soap and water	Not at all	Rarely	Neutral	Somewhat	Largely
Cough and sneeze on the inside of your elbow	Not at all	Rarely	Neutral	Somewhat	Largely
Use of tissue papers	Not at all	Rarely	Neutral	Somewhat	Largely
Not shaking hands	Not at all	Rarely	Neutral	Somewhat	Largely
Staying home if you get a cold	Not at all	Rarely	Neutral	Somewhat	Largely
Keep 1.5 meters (two arm's lengths) distance	Not at all	Rarely	Neutral	Somewhat	Largely
Stay at home as much as possible	Not at all	Rarely	Neutral	Somewhat	Largely
Group formation in public spaces is prohibited (3 or more people who do not form a household)	Not at all	Rarely	Neutral	Somewhat	Largely

The gluten free diet

With these questions we want to learn how you and your child deal with the gluten-free diet in daily life. Complete the questions below for the period **before the start of the covid-19 pandemic**. Questions concerning the current situation will follow later below this section.

11. In addition to the child that is the subject of this questionnaire, how many family members have a diagnosis of celiac disease?

    …………………………………………………………………………………………………..
    Have the other family members been diagnosed by a doctor?
    a. ☐ Yes
    b. ☐ No
12. How many family members are on a strict gluten-free diet besides your child?......................

You and the gluten free diet

13. Does your child follow a gluten free diet?
    a. ☐ Yes

b. ☐ No

If the answer is no, skip ahead to question number. 24

14. Does your child intentionally eat gluten?
    a. ☐ Yes
    b. ☐ No

If yes, in which of the following situations does your child intentionally eat gluten? (Check appropriate answers)

☐ At home
☐ If he/she is with other family members (such as grandparents)
☐ If he/she is with friends
☐ At special occasions (birthday, party, etc.)
☐ When eating out (at a restaurant for example)
☐ At school/work
☐ During sport activities
☐ During camp
☐ During holidays

15. If my child eats elsewhere, we discuss the gluten-free diet with the person providing the meal for my child.
    a. ☐ Yes
    b. ☐ No
16. We store the gluten-containing products separately from the gluten-free products.
    a. ☐ Yes
    b. ☐ No
17. Other people who do not follow the gluten-free diet can use the gluten-free butter and spreads.
    a. ☐ Yes
    b. ☐ No
18. If necessary, gluten free food is prepared with a separate toaster, bread box, fryer, baking pan.
    a. ☐ Yes
    b. ☐ No
19. My child eats gluten-free bread, bought from a local bakery that makes the bread themselves.
    a. ☐ Yes
    b. ☐ No
20. My child eats food that contains wheat starch.
    a. ☐ Yes
    b. ☐ No
21. My child eats food labeled "may contain traces of gluten or wheat".
    a. ☐ Yes
    b. ☐ No
22. My child eats food labeled "prepared in a gluten / wheat processing environment".
    a. ☐ Yes
    b. ☐ No

23. If my child has to use medicine, we make sure they are gluten-free.
    a. ☐ Yes
    b. ☐ No

The gluten free diet during the COVID-19 outbreak

24. Since the start of the coronavirus outbreak in the Netherlands (23.02.2020), has your child eaten anything that contains gluten that you know about?
    a. ☐ Yes I am sure my child has eaten gluten, this was intentional
    b. ☐ Yes I am sure my child has eaten gluten on accident
    c. ☐ Yes, I think my child has eaten gluten.
    d. ☐ No, I am sure my child did not eat gluten
    e. ☐ No, I think my child did not eat gluten
25. Have you been able to buy enough gluten-free products since the start of the coronavirus outbreak?
    a. ☐ Yes, we could buy the same gluten-free products / ingredients as we always do
    b. ☐ No, not all gluten-free products / ingredients that we usually buy were available, but there were plenty of alternative products available
    c. ☐ No, there were not enough gluten-free options available
26. Have you taken measures to ensure that you do not encounter problems following the gluten-free diet during this time?
    a. ☐ Yes
    b. ☐ No
27. If yes, how did you ensure that your child did not have problems following the gluten-free diet?

    ………
    ………
    ………
    ………………………………………………..
28. During this period, have there been any problems that you encountered with regards to your child's gluten-free diet? (multiple answer options are possible, check what is applicable and add other factors if necessary)
    a. ☐ Less availability of products
    b. ☐ A limited possibility to buy gluten-free products from abroad
    c. ☐ Provision of information about gluten-free products such as clear labels in online supermarkets or other shops
    d. ☐ Other, namely……
    …………
29. Are there any benefits or things that are easier now than before with regards to following the gluten-free diet? (multiple answer options are possible, check what is applicable and add other factors if necessary)
    a. ☐ Shopping online makes gluten-free choices easier
    b. ☐ Because we are more at home now, following the diet is easier

c. ☐ Other, namely………

30. Would you like to receive support from your child's healthcare provider or others during this time of the corona outbreak?
    a. ☐ Yes
    b. ☐ No

If answer yes, in what form? Can you give an example?
…….

31. Has anything changed with regard to your child's celiac disease during the period of the covid-19 outbreak, which you would like to persist after the COVID-19 pandemic? This could be, for example, with regards to the gluten-free diet, or the care your child receives from doctors, dieticians or other care providers.
    a. ☐ Yes
    b. ☐ No

If answer yes, in what form? Can you give an example?
…….

Nutrition before implementation of COVID-19 measurements

We will ask a couple of questions about your child's diet (how and what he/she eats). First we want to know something about what this was like before the corona period. And later we will ask you how this changed when the corona measures were introduced.

We would like to ask you to answer the following questions about your child's diet. These questions refer to a normal, average school week (before the implementation of COVID-19 measures).

32. How many days a week does your child eat breakfast? 1 | 2 | 3 | 4 | 5 | 6 | 7
33. How many days a week does your child eat lunch? 1 | 2 | 3 | 4 | 5 | 6 | 7
34. How many days a week does your child eat dinner? 1 | 2 | 3 | 4 | 5 | 6 | 7
35. How many days a week does your child eat fruit? 1 | 2 | 3 | 4 | 5 | 6 | 7
    How many pieces of fruit does your child eat on average per day?
    - ☐Less than 1 piece
    - ☐1 piece
    - ☐2 pieces
    - ☐More than 2 pieces
36. How many days a week does your child eat vegetables? 1 | 2 | 3 | 4 | 5 | 6 | 7
    How big is the portion of vegetables?
    - ☐ Less than 1 serving spoon

- ☐1 serving spoon
- ☐2 serving spoons
- ☐3 serving spoons
- ☐More than 3 serving spoons

37. Circle below how much of the following your child drinks per day **(before the implementation of COVID-19 measures).**

Sugared drinks (soft drinks, syrup, sports drinks)	Not applicable	1-2 glasses	3-4 glasses	5-6 glasses	More than 6 glasses
Fruit juice (such as orange juice and apple juice)	Not applicable	1-2 glasses	3-4 glasses	5-6 glasses	More than 6 glasses
Diet drinks (such as 0% syrup and diet soda)	Not applicable	1-2 glasses	3-4 glasses	5-6 glasses	More than 6 glasses
Water	Not applicable	1-2 glasses	3-4 glasses	5-6 glasses	More than 6 glasses
Skim milk	Not applicable	1-2 glasses	3-4 glasses	5-6 glasses	More than 6 glasses
Semi-skimmed milk	Not applicable	1-2 glasses	3-4 glasses	5-6 glasses	More than 6 glasses
Whole milk	Not applicable	1-2 glasses	3-4 glasses	5-6 glasses	More than 6 glasses
low-fat/fat-free chocolate milk	Not applicable	1-2 glasses	3-4 glasses	5-6 glasses	More than 6 glasses
Chocolate milk, whole	Not applicable	1-2 glasses	3-4 glasses	5-6 glasses	More than 6 glasses

38. How many days a week do you cook at home? 1 | 2 | 3 | 4 | 5 | 6 | 7
39. How many days a week do you eat out? 1 | 2 | 3 | 4 | 5 | 6 | 7
40. How many days a week do you order take out? 1 | 2 | 3 | 4 | 5 | 6 | 7

41. Which meals did your child eat on average on a weekday (breakfast, lunch, dinner)?
    a. ☐Breakfast
    b. ☐Lunch
    c. ☐Dinner
42. How many times did your child eat a healthy snack on a weekday? (such as fruits, nuts, vegetables, sandwich)

    ………
43. How many times did your child eat an unhealthy snack on a weekday? (such as a biscuit, candy, or chips)

………
………………..

Nutrition during the COVID-19 measures

We would like to ask you to complete the following questions about your child's diet during the COVID-19 outbreak (after 23/02/2020).

44. How many days a week does your child eat breakfast? 1 | 2 | 3 | 4 | 5 | 6 | 7
45. How many days a week does your child eat lunch? 1 | 2 | 3 | 4 | 5 | 6 | 7
46. How many days a week does your child eat dinner? 1 | 2 | 3 | 4 | 5 | 6 |7
47. How many days a week does your child eat fruit? 1 | 2 | 3 | 4 | 5 | 6 | 7

How many pieces of fruit does your child eat on average per day?

- ☐Less than 1 piece
- ☐1 piece
- ☐2 pieces
- ☐More than 2 pieces

48. How many days a week does your child eat vegetables? 1 | 2 | 3 | 4 | 5 | 6 | 7

How big is the portion of vegetables?

- ☐ Less than 1 serving spoon
- ☐1 serving spoon
- ☐2 serving spoons
- ☐3 serving spoons
- ☐More than 3 serving spoons

49. Circle below how much of the following your child drinks per day

Sugared drinks (soft drinks, syrup, sports drinks)	Not applicable	1-2 glasses	3-4 glasses	5-6 glasses	More than 6 glasses
Fruit juice (such as orange juice and apple juice)	Not applicable	1-2 glasses	3-4 glasses	5-6 glasses	More than 6 glasses
Diet drinks (such as 0% syrup and diet soda)	Not applicable	1-2 glasses	3-4 glasses	5-6 glasses	More than 6 glasses
Water	Not applicable	1-2 glasses	3-4 glasses	5-6 glasses	More than 6 glasses

Skim milk	Not applicable	1-2 glasses	3-4 glasses	5-6 glasses	More than 6 glasses
Semi-skimmed milk	Not applicable	1-2 glasses	3-4 glasses	5-6 glasses	More than 6 glasses
Whole milk	Not applicable	1-2 glasses	3-4 glasses	5-6 glasses	More than 6 glasses
low-fat/fat-free chocolate milk	Not applicable	1-2 glasses	3-4 glasses	5-6 glasses	More than 6 glasses
Chocolate milk, whole	Not applicable	1-2 glasses	3-4 glasses	5-6 glasses	More than 6 glasses

50. How many days a week do you cook at home? 1 | 2 | 3 | 4 | 5 | 6 | 7
51. How many days a week do you eat out? 1 | 2 | 3 | 4 | 5 | 6 | 7
52. How many days a week do you order take out? 1 | 2 | 3 | 4 | 5 | 6 | 7

Experiences eating pattern:

53. Compared to a normal school week, your child ate ……… during the past week (circle if applicable)

Breakfast much less often / less often / just as often / more often / a lot more often
Lunch much less often / less often / just as often / more often / a lot more often
Dinner much less often / less often / just as often / more often / a lot more often
Unhealthy snacks (such as cookies, chips, bars, chocolate, candy)
much less often / less often / just as often / more often / a lot more often
Healthy snacks (such as nuts, fruits, vegetables, a sandwich)
much less often / less often / just as often / more often / a lot more often
Fruit / vegetablesmuch less often / less often / just as often / more often / a lot more often
Dairy products much less often / less often / just as often / more often / a lot more often
Sugared drinks much less often / less often / just as often / more often / a lot more often

The following questions are about your child's eating habits. By habits we mean what and how much someone has eaten.

54. Have the eating habits in your family changed compared to a normal school week?
    a. ☐ Yes
    b. ☐ No

If yes, have you consciously made adjustments in your family's eating habits compared to a normal school week?
    c. ☐ Yes
    d. ☐ No

If yes, how have the eating habits changed?
………
……………………

………
……………………..

55. My child's diet this week compared to a normal school week is:

Much less healthy | more unhealthy | equally as healthy/unhealthy | healthier | much healthier

56. How much influence did the following factors have on your child's eating habits last week? (circle if applicable)

My child's stress, anxiety and worries not at all | little | not little, not much | much | very much

Stress, anxiety and concerns of other family members not at all | little | not little, not much | much | very much

Boredom of my child not at all | little | not little, not much | much | very much

Different day structure not at all | little | not little, not much | much | very much

My child's health complaints not at all | little | not little, not much | much | very much

Health complaints of other family members not at all | little | not little, not much | much | very much

Financial worries (worries about money at home) not at all | little | not little, not much | much | very much

Desire to build up good resistance not at all | little | not little, not much | much | very much

Availability of products in the store not at all | little | not little, not much | much | very much

Other family members buying groceries not at all | little | not little, not much | much | very much

My child does not buy snacks / food at school now not at all | little | not little, not much | much | very much

57. How many main meals (breakfast, lunch, dinner) did your child eat on average on a weekday?
    - ☐none
    - ☐One
    - ☐Two
    - ☐Three
58. Is this in your opinion
    - ☐much less than
    - ☐less than
    - ☐equally as much
    - ☐more than
    - ☐much more than

    Normally before the COVID-19 measurements?

59. How many times did your child eat a healthy snack (such as a handful of nuts, a piece of fruit, raw vegetables, a sandwich) besides the main meals <u>on a weekday?</u>
    a. ☐Zero

b. ☐One
c. ☐Two
d. ☐Three
e. ☐More than three

60. Is this in your opinion
    - ☐much less than
    - ☐less than
    - ☐equally as much
    - ☐more than
    - ☐much more than

    Normally before the COVID-19 measurements?

61. How many times did your child eat an unhealthy snack (such as cookies, chips, bars, chocolate, candy) besides the main meals, on a weekday?
    a. ☐Zero
    b. ☐One
    c. ☐Two
    d. ☐Three
    e. ☐More than three

62. Is this in your opinion
    - ☐much less than
    - ☐less than
    - ☐equally as much
    - ☐more than
    - ☐much more than

    Normally before the COVID-19 measurements?

Length and weight

63. Do you have a working scale at home?
    a. ☐ Yes
    b. ☐ No

    If yes, what is the current weight of your child? ………. Kg

It is best to weigh without clothing. Do you prefer to weigh with clothes on? This is also allowed.

64. Has a healthcare provider recently measured the height of your child (pediatrician, general practitioner, health clinic or the like)?
    a. ☐ Yes
    b. ☐ No

    If yes, specify:

Date: …………………………………………………….

Measured by: …………………………………..

Length in cm: ……………………………………........
