# Supplementary material for: Celiac Disease and the Gluten Free Diet during the COVID-19 Pandemic: Experiences of Children and Parents
Source: Medicina (Kaunas). 2023 Feb 21;59(3):425. doi: 10.3390/medicina59030425 (PMC10059717; doi:10.3390/medicina59030425)
Supplement: Supplementary file 1 [file medicina-59-00425-s001.zip › Supplementary Table S1.pdf]

medicina

MDPI

Supplementary Materials

Celiac Disease and the Gluten Free Diet during the COVID-19 Pandemic: Experiences of Children and Parents

Supplementary Table S1. Symptoms that occurred or worsened since start of the COVID-19 pandemic either related to celiac disease (CD) activity, a possible COVID-19 infection or both (concordant symptoms).

Symptom	Associated with COVID-19 or CD
Common cold	COVID-19
Coughing	COVID-19
Stuffy feeling	COVID-19
Shortness of breath or wheezing	COVID-19
Sore throat	COVID-19
Fever	COVID-19
Sneezing	COVID-19
Coughing up mucus	COVID-19
Fatigue	COVID-19 and CD (concordant symptom)
Muscle soreness	COVID-19
Chest pain	COVID-19
Diarrhea	COVID-19 and CD (concordant symptom)
Stomach ache	COVID-19 and CD (concordant symptom)
Nausea/ vomiting	COVID-19 and CD (concordant symptom)
Eye infection	COVID-19
Lost sense of smell	COVID-19
Headache	COVID-19 and CD (concordant symptom)
Constipation	CD
Bloated stomach/ flatulence	CD
Weight loss	CD
Lower energy level	CD
Red itchy rash	CD
Sores in mouth	CD
